# Impact of Continuous Positive Airway Pressure Therapy on Gastroesophageal Reflux Disease in Patients With Obstructive Sleep Apnea: A Prospective Cohort Study

**DOI:** 10.7759/cureus.90927

**Published:** 2025-08-25

**Authors:** Sohini Saha, Meghanad Meher

**Affiliations:** 1 Department of General Medicine, Institute of Medical Sciences and Siksha 'O' Anusandhan Medical Hospital, Bhubaneswar, IND

**Keywords:** continuous positive airway pressure, gastroesophageal reflux disease, obstructive sleep apnea, polysomnography, prospective

## Abstract

Introduction: This study aimed to evaluate the effects of continuous positive airway pressure (CPAP) therapy on gastroesophageal reflux disease (GERD) symptom severity and obstructive sleep apnea (OSA) severity in patients diagnosed with both conditions over a one-month period, using validated tools to examine CPAP adherence and adverse events.

Materials and methods: This prospective, single-center cohort study was conducted at the Department of Respiratory Medicine between May 2024 and March 2025. Eighty-nine adults aged 18-75 years with moderate-to-severe OSA (apnea-hypopnea index as AHI ≥ 15 events/h) confirmed by polysomnography and GERD diagnosed via clinical symptoms or endoscopic evidence were enrolled. The exclusion criteria included prior CPAP use, severe comorbidities, and inability to tolerate CPAP use. Baseline assessments included the Gastroesophageal Reflux Disease Questionnaire (GERD-Q) for GERD symptoms, Epworth Sleepiness Scale (ESS) for daytime sleepiness, and polysomnography for AHI, peripheral oxygen saturation (SpO_2_), and oxygen desaturation index (ODI). The patients received auto-titrating CPAP therapy with telemedicine support. Follow-up at one month included repeat GERD-Q, ESS, polysomnography, and CPAP adherence data (defined as >4 h/night on ≥70% of nights). Statistical analyses were performed using the Mann-Whitney U test, mediation analysis, and multivariable linear regression analysis, with significance set at p < 0.05.

Results: The study included 89 patients, with 55 male patients (61.8%) and 34 (38.2%) female patients. Of the 89 patients (mean age 55.04 ± 6.48 years, mean body mass index 31.84 ± 2.5 kg/m²), 72 (80.9%) achieved adequate CPAP adherence. Adequate adherence significantly reduced the GERD-Q scores (p < 0.05), AHI (p < 0.05), ODI (p < 0.05), and ESS scores (p < 0.05), with no significant change in SpO_2_ (p = 0.393). No adverse events were noted. Smoking and right-side/supine sleep positions negatively predicted GERD improvement, whereas the ESS reduction was a positive predictor. Mediation analysis showed that CPAP's effect on GERD was independent of changes in AHI, ODI, or ESS.

Conclusion: Adequate CPAP adherence significantly improved GERD and OSA severity without adverse effects, supporting its use as the primary therapy for patients with both conditions. Targeted interventions addressing smoking and sleep positions may further enhance these outcomes.

## Introduction

Obstructive sleep apnea (OSA) is a prevalent sleep-related breathing disorder characterized by intermittent complete or partial obstruction of the upper airway, leading to episodic hypoxemia and disruption of sleep continuity [1]. OSA and gastroesophageal reflux disease (GERD) are prevalent chronic conditions that frequently coexist, and studies have suggested a bidirectional relationship mediated by mechanisms such as increased intra-abdominal pressure, transient lower esophageal sphincter relaxation, and nocturnal arousal events [2,3]. During nocturnal hours, there are instances of prolonged gastric emptying, significantly protracted esophageal clearance, and a pronounced decrease in upper esophageal sphincter pressure [4]. OSA is correlated with a notable prevalence of GERD, where the regurgitated gastric contents in response to the onset of OSA may instigate inflammation of the upper airway and potentially result in obstruction [2-4]. The relationship between OSA and GERD remains controversial, with prior investigations demonstrating limitations and inconsistencies in their findings [3].

Continuous positive airway pressure (CPAP) therapy, the gold standard treatment for OSA, has been shown to improve sleep quality, reduce the apnea-hypopnea index (AHI), and enhance the overall quality of life (QoL) in patients with OSA [5]. Emerging evidence indicates that CPAP may also alleviate GERD symptoms by stabilizing airway pressure and reducing nocturnal reflux events, potentially improving esophageal acid exposure and symptom severity [2,6]. However, the precise effect of CPAP on GERD symptoms and QoL in patients with both conditions remains unclear, particularly in prospective observational settings. Ozcelik et al. [7] reported no improvement in GERD symptoms in OSA patients treated with CPAP.

Despite the growing body of literature, significant research gaps remain. Most studies have focused on retrospective analyses or small-scale interventional trials, with limited attention paid to prospective, real-world observational data capturing the dual effects of CPAP on OSA and GERD outcomes [3]. The lack of standardized GERD symptom assessment and long-term follow-up in prior research limits our understanding of CPAP’s sustained effects. Additionally, there is a paucity of data on how CPAP adherence influences GERD symptom improvement, a critical factor given variable patient compliance [8].

This study addressed these gaps by prospectively evaluating the effects of CPAP therapy on GERD symptom severity and OSA severity in a cohort of patients with both conditions using validated tools and a one-month follow-up period to capture real-world outcomes. This study aimed to evaluate the effects of CPAP therapy on GERD symptom severity and OSA severity in patients diagnosed with both OSA and GERD over a one-month period. Specifically, this study sought to assess the change in GERD symptom severity using a validated questionnaire to assess scores from baseline to one month following CPAP therapy initiation. We also aimed to evaluate the change in OSA severity, measured by the AHI via polysomnography (PSG), over the same period. Additionally, we examined CPAP adherence, defined as the average nightly use in hours, and explored its association with improvements in GERD symptoms. Finally, this study documented the frequency and nature of adverse events related to CPAP use, such as nasal irritation or mask discomfort, to provide a comprehensive understanding of the impact of therapy over the study period.

## Materials and methods

This prospective, non-randomized, cohort, single-center observational study included consecutive patients who visited the Department of Respiratory Medicine between May 2024 and March 2025. Ethical approval was obtained from the Institutional Ethics Committee (IEC/IMS.SH/SOA/2024/745). All enrolled patients provided written informed consent, and the study was conducted in accordance with the principles of the Declaration of Helsinki. The consent process involved a detailed explanation of the study's purpose, procedures, potential risks, and benefits. The participants were informed of their right to withdraw from the study at any time without affecting their clinical care.

**Figure 1:**
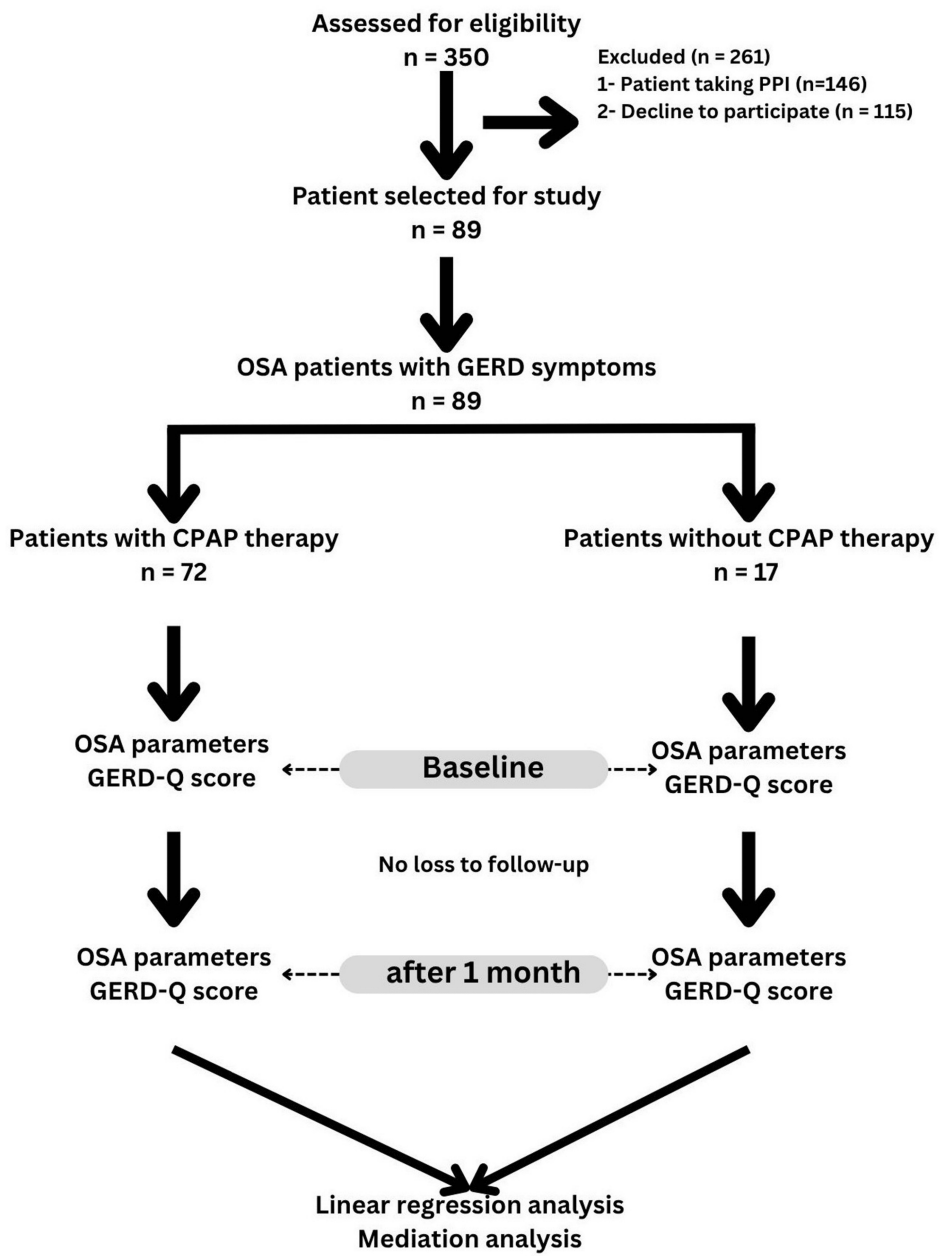
Study flow chart. PPI: proton pump inhibitors; OSA parameters (AHI: apnea-hypopnea index, SpO_2_: peripheral capillary oxygen saturation, ESS: Epworth sleepiness scale, and ODI: Oxygen desaturation index); GERD-Q: gastroesophageal reflux disease questionnaire; GERD: gastroesophageal reflux disease; CPAP: continuous positive airway pressure.

The study design is shown in Figure 1. An observational design was chosen because of ethical constraints, as withholding CPAP therapy from patients diagnosed with OSA would have been inappropriate, and randomization to a sham CPAP control group was deemed unfeasible in this context. This study was conducted at a tertiary care sleep clinic affiliated with the university hospital. The clinic provided access to PSG facilities, CPAP equipment, and gastroenterology expertise for GERD assessment. Patients were recruited from the sleep clinic population following routine diagnostic PSG.

Eligible participants were adults aged 18-75 years with a confirmed diagnosis of moderate-to-severe OSA (AHI ≥ 15 events/h) via PSG using a PSG machine (Alice A6, Philips, USA) multi-channel sleep monitor [9] and a confirmed diagnosis of GERD based on clinical symptoms (heartburn and regurgitation) and/or endoscopic evidence of esophagitis [10]. Participants were required to initiate CPAP therapy as part of the standard care for OSA and to provide informed consent and comply with the study follow-up. The exclusion criteria included prior CPAP use or other OSA treatments (such as surgery and oral appliances); severe comorbidities such as cardiovascular pathologies, diabetes, cerebrovascular disease, renal or hepatic disorders, dominant central sleep apnea, respiratory ailments other than OSA such as chronic obstructive pulmonary disease, or active malignancy; use of medications known to significantly affect GERD (such as high-dose proton pump inhibitors as PPIs); pregnancy or planned pregnancy during the study period; and inability to tolerate CPAP therapy during the initial titration phase.

The sample size was determined using G*Power software version 3.6.9 (Heinrich-Heine-Universität Düsseldorf, Düsseldorf, Germany) based on an effect size of 0.46 derived from a prior study examining CPAP’s effect on GERD in patients with OSA. With 90% power (β = 0.10), 5% alpha error (two-tailed), and 95% confidence interval, the analysis indicated a required sample size of 75 participants to detect statistically significant differences between groups [11]. A 20% drop-out of the obtained sample size (n = 89) aligned with the parameters, ensuring robust detection of clinically meaningful changes in GERD symptoms after CPAP therapy.

Patients were identified using the sleep clinic database following routine PSG to confirm the diagnosis of OSA. Patients with a concurrent GERD diagnosis [10] verified by a gastroenterologist were invited to participate in the study. At baseline, prior to CPAP initiation, participants underwent PSG to confirm the AHI and determine the severity of OSA. GERD symptoms were assessed using the GERD Questionnaire (GERD-Q), a validated tool for measuring the frequency and severity of heartburn and regurgitation, used under license number 6080241127551 for research purposes [12]. The following questions were evaluated: burning sensation behind the breastbone (heartburn), regurgitation of stomach contents into the throat or mouth, pain in the upper central stomach area, nausea, sleep disturbances due to heartburn or regurgitation, and prior use of over-the-counter medications for heartburn or regurgitation. Each item was scored based on the symptom frequency per week, with response options as follows: 0 (0 days), 1 (1 day), 2 (2-3 days), and 3 (4-7 days). The scores for all six items were summed for each participant, and a total score of ≥ 8 was used to diagnose GERD, consistent with prior studies demonstrating high sensitivity and specificity for identifying esophagitis and excluding functional heartburn [13,14].

The study also employed the Epworth Sleepiness Scale (ESS) to assess excessive daytime sleepiness, a key indicator of OSA [15]. Permission was obtained to use the ESS questionnaire. The ESS consists of eight questions that evaluate the likelihood of dozing off or falling asleep in various daily situations, such as sitting and reading, watching television, or being a passenger in a car. Each item was scored on a scale of 0 (no chance of dozing) to 3 (high chance of dozing), with a total score ranging from 0 to 24. A score of ≥10 was used as the threshold to identify individuals with excessive daytime sleepiness suggestive of OSA, consistent with established clinical guidelines. To ensure an accurate assessment, patients were instructed to complete the ESS based on their typical sleep patterns, and their responses were collected under standardized conditions to minimize external influences.

At the initial visit, all participants involved in the study were provided with a uniform educational program pertaining to optimal sleep practices, encompassing recommendations for establishing a consistent sleep routine; abstention from alcoholic beverages, smoking, and sedative use during evening hours; and the maintenance of a sleep duration ranging from 7 to 8 h per night, in addition to guidelines for adhering to a nutritious diet, engaging in regular physical activity, and following prescribed medication regimens. Demographic details, such as age, sex, medical history, use of medications, and history of smoking or alcohol consumption, were noted. The body mass index (BMI) was calculated as weight divided by height squared (kg/m^2^). To minimize bias from symptom suppression, all patients were instructed to refrain from using PPIs during the study period, with adherence monitored through self-reporting and follow-up assessments.

Adherence to CPAP therapy was defined as the use of the device for > 4 h per day on at least 70% of the monitored days [16]. All patients received CPAP treatment using an auto-titrating CPAP machine (pressure 4-20 cm H2O, AirSense™ 11 Auto CPAP, ResMed Inc., San Diego, USA). Following the commencement of CPAP therapy administered by a trained research nurse, additional assistance was provided through a web-based telemedicine platform (AirView; ResMed Inc., San Diego, California, USA). Telemedicine data were meticulously evaluated by a specially trained research nurse who assessed adherence, mask leakage, and residual apneas on a weekly basis and initiated telephone communication as deemed necessary.

Follow-up assessments were conducted at one-month post-CPAP initiation, which included repeat GERD-Q to assess changes in GERD symptoms, repeat PSG to measure changes in AHI, peripheral oxygen saturation (SpO_2_), and oxygen desaturation index (ODI) as determined by PSG examination. CPAP adherence data were downloaded from the device, and documentation of any adverse events or changes in health status was noted.

Data were collected using secure electronic case report forms and stored in a password-protected database compliant with the Health Insurance Portability and Accountability Act (HIPAA) regulations. Only de-identified data were used for the analysis to ensure participant confidentiality. The primary outcome was the change in GERD symptom severity, measured using the GERD-Q score from baseline to one month. Secondary outcomes included changes in OSA severity, measured by AHI (events/hour), SpO_2_, and ODI on PSG from baseline to one month, CPAP adherence defined as average nightly use (hours/night) over a one-month period, and the frequency of adverse events related to CPAP use, such as nasal irritation or mask discomfort.

Statistical analysis

Data were analyzed using IBM SPSS Statistics for Windows, Version 20 (Released 2011; IBM Corp., Armonk, New York, United States). Categorical data are presented as frequencies and percentages. Continuous data (AHI, ESS, ODI, SpO2, and GERD-Q score) were checked for normal distribution using the Shapiro-Wilk test and confirmed as non-normal distribution by a Q-Q plot. Continuous data are presented as the mean, median, and interquartile range. Baseline data were compared between groups (CPAP-adherent and partially adherent) using the Mann-Whitney U test (non-parametric test). The efficacy of CPAP therapy was evaluated by comparing changes in outcome variables after one month using the Mann-Whitney U test and mediation analysis. Multivariable linear regression analysis was performed to identify predictors of the GERD-Q score. Statistical significance was set at p < 0.05.

## Results

No adverse effects related to CPAP therapy, such as nasal irritation or mask discomfort, were reported by the participants during the one-month study period. The use of auto-titrating CPAP machines and weekly telemedicine monitoring likely contributed to the absence of notable adverse events in this study.

The study included 89 participants, of whom 55 (61.8%) were men and 61 (68.5%) were non-smokers. Alcohol consumption was reported by 32 (36.0%) participants, and 61 (68.5%) participants had moderate OSA. Most participants preferred sleeping on the right side, and CPAP adherence was high, as observed in 72 (80.9%) participants. The baseline characteristics revealed a male-predominant cohort with low smoking rates but notable alcohol use. The high prevalence of moderate OSA severity suggests the need for targeted interventions in this subgroup (Table 1).

**Table 1:** Baseline characteristics of the study sample. OSA: Obstructive sleep apnea; AHI: Apnea-hypopnea index; CPAP: Continuous positive airway pressure. Data are presented as frequency (n) and percentage (%), where n denotes the number of participants.

Variables	Category	n	%
Sex	Male	55	61.8
	Female	34	38.2
Smoking	Yes	28	31.5
	No	61	68.5
Alcohol	Yes	32	36.0
	No	61	68.5
OSA based on AHI	Moderate OSA (AHI of 15–29.9 events per hour)	61	68.5
	Severe OSA (AHI of ≥30 events per hour)	28	31.5
Sleep position	Left	21	23.6
	Right	47	52.8
	Supine	21	23.6
CPAP adherence	Adequate	72	80.9
	Partial	17	19.1

The participants had a mean age of 55.04 ± 6.48 years, with male participants slightly older than female participants. The study population had a mean BMI of 31.84 ± 2.5 kg/m², corresponding to Class 1 obesity (BMI 30-34.9 kg/m²) [17]. The median AHI was 24 events/h, confirming moderate-to-severe OSA, while the ESS score was low, suggesting mild daytime sleepiness. Mean SpO₂ was near-normal (94.99%, SD = 0.82), but the ODI was elevated (median = 28, IQR = 4), reflecting significant nocturnal hypoxia. The median GERD-Q score was 12 (IQR: 2), indicating frequent reflux symptoms. The cohort had a high burden of obesity and moderate-to-severe OSA with notable nocturnal hypoxia despite near-normal SpO₂ (Table 2).

**Table 2:** Baseline characteristics of the study sample. OSA: Obstructive sleep apnea; AHI: Apnea-hypopnea index; BMI: Body mass index; SpO₂: Peripheral capillary oxygen saturation; GERD-Q: Gastroesophageal Reflux Disease Questionnaire; ESS: Epworth Sleepiness Scale. Data are presented as mean and standard deviation (SD) with median and interquartile range (IQR).

Variables		Mean	SD	Median	IQR
Age (years)	Female	54.70	6.55	54.00	11.5
	Male	55.25	6.49	55.00	12
	Overall	55.04	6.48	55.00	12
BMI	kg/m^2^	31.84	2.50	31.40	4.2
AHI	Events/hour	24.58	5.62	24.00	9
ESS	Score	7.51	2.32	7.00	3
SpO_2_	Percentage	94.98	0.81	95.00	2
ODI	Events/hour	28.09	2.92	28.00	4
GERD-Q score	Score	11.42	1.66	12.00	2

The Mann-Whitney U test revealed no significant differences between the partial CPAP adherent (n = 17) and adequate CPAP (n = 72) adherent groups in the key sleep-related parameters. The AHI was comparable between the groups (p = 0.746). Similarly, ESS scores (p = 0.628) and SpO₂ (p = 0.293) did not differ significantly between the two groups. The ODI (p = 0.551) and GERD-Q scores (p = 0.867) did not differ significantly between the groups (Table 3).

**Table 3:** Comparison of baseline data between study groups by the Mann-Whitney U test. p > 0.05 denotes no statistical significance. AHI: Apnea-hypopnea index; SpO₂: Peripheral capillary oxygen saturation; GERD-Q: Gastroesophageal Reflux Disease Questionnaire; ESS: Epworth Sleepiness Scale; ODI: Oxygen desaturation index. Data are presented as mean and standard deviation (SD) with median and interquartile range (IQR), where n denotes the number of participants in each group.

Variables	Partial CPAP adherence (n = 17, 19%)			Adequate CPAP adherence (n = 72, 81%)			U stats	p-value	Effect size
	Mean	SD	Median (IQR)	Mean	SD	Median (IQR)			
AHI (events/hour)	24.59	3.58	25 (3)	24.58	6.02	23.5 (11)	643.5	0.746	0.05
ESS	7.59	1.27	8 (1)	7.50	2.51	7 (4.2)	658.5	0.628	0.07
SpO_2_ (%)	95.18	0.72	95 (1)	94.94	0.83	95 (2)	707.5	0.293	0.15
ODI	27.88	1.61	28 (2)	28.13	3.15	27.5 (6)	555.5	0.551	0.02
GERD-Q score	11.29	1.21	12 (1)	11.45	1.76	12 (2.2)	628.5	0.867	-0.09

The Mann-Whitney U test revealed significant differences in treatment responses between the partial CPAP (n = 17) and adequate CPAP (n = 72) adherent groups. The adequate CPAP adherent group demonstrated substantially greater improvements in AHI, ESS, ODI, and GERD-Q scores, with large effect sizes across all significant outcomes. Only the SpO₂ improvement showed no significant difference between the groups (p = 0.393, r = - 0.13). The consistent pattern of results indicated that adequate CPAP adherence was markedly more effective than partial CPAP adherence in reducing OSA severity, daytime sleepiness, SpO_2_, and GERD symptoms, with nearly all comparisons showing large effect sizes (Table 4).

**Table 4:** Comparison of change in outcome variables between study groups by the Mann-Whitney U test. p < 0.05 denotes statistical significance. AHI: Apnea-hypopnea index; SpO₂: Peripheral capillary oxygen saturation; GERD-Q: Gastroesophageal Reflux Disease Questionnaire; ESS: Epworth Sleepiness Scale; ODI: Oxygen desaturation index. Data are presented as mean and standard deviation (SD) with median and interquartile range (IQR), where n denotes number of participants in each group.

Variables	Partial CPAP adherence (n = 17, 19%)			Adequate CPAP adherence (n = 72, 81%)			U stats	p-value	Effect size
	Mean	SD	Median (IQR)	Mean	SD	Median (IQR)			
AHI (events/hour)	-1.70	2.02	-2(1)	-6.36	2.15	-6(2.2)	1141	0.001*	0.86
ESS	-0.41	1.00	-1(1)	-3.93	2.51	-4(4)	1113	0.001*	0.81
SpO_2_ (%)	1.35	0.86	1(1)	1.55	0.97	2(1)	529	0.393	-0.13
ODI	-3.24	2.54	-3(2)	-17.33	3.51	-17(5)	1224	0.001*	1.00
GERD-Q score	0.76	1.34	1(2)	-4.26	1.90	-4(3)	1206	0.001*	0.97

Linear regression analysis identified several significant predictors of improvement in GERD. Smoking status, right-sided sleep position, and supine sleep position were negatively associated with GERD improvement, suggesting that these factors may hinder the therapeutic response. A greater reduction in ESS scores predicted better GERD outcomes, whereas a higher ODI was paradoxically linked to worsening symptoms. Changes in BMI, sex, and AHI did not significantly influence GERD improvement (P > 0.05). The model highlights modifiable factors (sleep position and smoking) and symptom profiles (sleepiness and hypoxia) as key determinants of the GERD treatment response in patients with OSA (Table 5).

**Table 5:** Multivariable linear regression analysis for gastroesophageal reflux disease (GERD) improvement. *p < 0.05 denotes statistical significance, AHI: Apnea-hypopnea index; ESS: Epworth Sleepiness Scale; BMI: Body mass index. To: Baseline, T1: After one-month, Ref: Reference variable.

Variables	Unstandardized Coefficients (B)	Standard Error	t value	p-value
					Sex (female) (Ref: male)	-0.61	0.53	-1.15	0.255
BMI (kg/m^2^)	-0.12	0.10	-1.23	0.224
Smoking (yes) (Ref: no)	-1.51	0.62	-2.43	0.018*
Sleep position (right) (Ref: left)	-1.42	0.60	-2.37	0.02*
Sleep position (supine) (Ref: left)	-2.16	0.84	-2.58	0.012*
AHI (T0-T1)	0.01	0.14	0.05	0.961
ESS (T0-T1)	-0.43	0.16	-2.67	0.009*
Oxygen Desaturation Index (T0-T1)	0.36	0.07	4.96	0.001*

Mediation analysis revealed a significant direct effect of CPAP therapy on GERD symptoms across all mediators (AHI, ESS, and ODI), with p = 0.001 for each (Table 6).

**Table 6:** Mediation analysis to assess the effect of CPAP on GERD. *p < 0.05 denotes statistical significance, AHI: Apnea-hypopnea index; ESS: Epworth Sleepiness Scale; ODI: Oxygen desaturation index; GERD: Gastroesophageal reflux disease; CPAP: Continuous positive airway pressure.

Mediator	Direct effect (p-value)	Indirect effect (p-value)	Total effect (p-value)
AHI (events/hour)	-4.92 (0.001*)	0.11 (0.935)	-5.03 (0.001*)
ESS	-4.83 (0.001*)	-0.25 (0.678)	-5.03 (0.001*)
ODI	-4.52 (0.001)	-0.49 (0.991)	-5.03 (0.001*)

**Figure 2:**
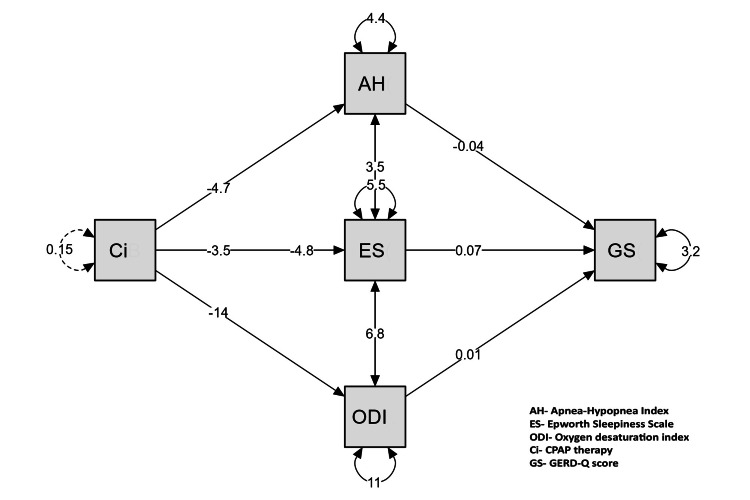
Mediation analysis demonstrating the effect of CPAP therapy (Ci) on GERD-Q score (GS) via the apnea-hypopnea index (AHI), Epworth Sleepiness Scale (ESS), and Oxygen Desaturation Index (ODI). Path coefficients and residual covariances are indicated on the diagram. The values −0.04, 0.07, 0.01, −4.7, −3.5, and −14 represent path coefficients, while 3.5, 4.8, and 6.8 represent residual covariances. This figure is derived from the study data.

However, indirect effects through AHI (p = 0.935), ESS (p = 0.678), and ODI (p = 0.991) were not statistically significant. The total effect of CPAP on GERD remained significant (p = 0.001), indicating that the improvement in GERD symptoms was primarily due to a direct effect of CPAP therapy rather than mediation through the selected sleep-related parameters (Figure 2).

## Discussion

The findings of this study demonstrated that adequate CPAP adherence significantly improved GERD symptoms, OSA severity, daytime sleepiness, and nocturnal hypoxia, as measured using validated tools such as the GERD-Q, AHI, ESS, and ODI. These results align with and expand upon the existing literature, offering a comprehensive evaluation of CPAP’s therapeutic impact over a one-month period in a real-world clinical setting.

The primary finding of this study was that adequate CPAP adherence (defined as >4 h per night on ≥70% of nights) resulted in significant reductions in GERD-Q scores, indicating improved GERD symptom severity. This is consistent with prior research, such as that of Green et al. [18], who reported that CPAP therapy reduces nocturnal GERD symptoms in patients with OSA by stabilizing the upper airway and reducing intrathoracic pressure fluctuations that exacerbate reflux. According to Tamanna et al. [8], a minimum compliance of 25% is needed to produce the positive effects of CPAP in patients with OSA and GERD. Our study revealed that 73 (81%) participants had adequate adherence to CPAP therapy. Qiao et al. [19] identified the following factors associated with a greater likelihood of long-term CPAP adherence: older age, BMI, presence of a bed partner, non-smoker status, presence of diabetes, presence of congestive heart failure, lack of depression, and compliance during the initial months of treatment. Our results indicated that adequate CPAP adherence led to significant improvements in GERD and OSA symptoms, which is consistent with previous studies [6,8,18].

The significant improvement in OSA severity, as evidenced by reductions in AHI and ODI, aligns with the well-established evidence of CPAP’s efficacy in treating OSA. For instance, Patel et al. [20] demonstrated that CPAP therapy significantly reduced the AHI in patients with moderate-to-severe OSA, thereby improving sleep architecture and oxygenation. The current study’s observation of a near-normal baseline SpO₂ (94.99%) but elevated ODI suggests that intermittent hypoxia, rather than sustained desaturation, is a critical factor in OSA-related morbidity, which CPAP effectively mitigates by CPAP. This finding is consistent with that of Javaheri et al. [21], who noted that CPAP reduced the ODI, thereby decreasing the cardiovascular risk associated with nocturnal hypoxia.

However, the lack of significant mediation of GERD improvement through changes in AHI, ESS, or ODI is a novel finding that contrasts with the findings of previous studies. For example, Shepherd et al. [2] suggested that CPAP’s effect on GERD is mediated by a reduction in OSA severity, as improved airway patency reduces negative intrathoracic pressure, which promotes reflux. The mediation analysis in the current study, which showed a direct effect of CPAP on GERD symptoms independent of AHI or ODI changes, suggests that mechanisms beyond OSA severity reduction, such as direct stabilization of the lower esophageal sphincter or reduced diaphragmatic stress, may play a role. This discrepancy could be due to differences in the study populations, as Shepherd et al. [2] included patients with mild OSA, whereas the current study focused on moderate-to-severe cases (AHI ≥ 15 events/h). Additionally, the one-month follow-up period may have been too short to capture full mediation effects, as extended treatment time has been reported to strengthen the association between AHI reduction and GERD improvement with CPAP [22].

The study also identified predictors of GERD improvement, with smoking status, right-side sleep position, and supine sleep position negatively associated with the therapeutic response. These findings are supported by those of Fujiwara et al. [23], who noted that smoking exacerbates GERD by weakening the lower esophageal sphincter and increasing acid exposure. The negative impact of the right and supine sleep positions may be explained by anatomical factors, as these positions increase the likelihood of acid reflux due to the gravitational effects on gastric contents, as reported by Khoury et al. [24]. Conversely, the positive association between ESS score reduction and GERD improvement suggests that alleviating daytime sleepiness may enhance patients’ ability to adhere to lifestyle modifications, such as dietary changes, which further reduce GERD symptoms.

The significant improvements in GERD and OSA outcomes among patients with adequate CPAP adherence can be attributed to several physiological mechanisms. CPAP therapy maintains positive airway pressure, prevents airway collapse, and reduces negative intrathoracic pressure swings, which promote acid reflux. This is particularly relevant in patients with OSA, where repetitive airway obstruction increases diaphragmatic pressure and facilitates the retrograde movement of gastric contents [5,6]. CPAP likely reduces these pressure changes by stabilizing the airway, as suggested by Ing et al. [25]. Additionally, improvement in sleep quality with CPAP, as reflected by reduced ESS scores, may indirectly benefit GERD by promoting better adherence to sleep hygiene practices, such as avoiding late-night meals, which exacerbate reflux. The lack of significant differences in baseline characteristics (such as AHI, ESS, SpO₂, ODI, and GERD-Q) between the adequate and partial CPAP adherent groups suggests that adherence, rather than baseline severity, drives the therapeutic outcomes. This underscores the importance of patient education and support, as provided in this study, through a uniform educational program and telemedicine.

This evidence highlights several key components of intervention strategies for enhancing CPAP adherence among individuals with OSA. Comprehensive patient education is vital and encompasses information on OSA, its diagnosis, symptoms, CPAP therapy, anticipated treatment outcomes, and daily management practices. Establishing clear goals for CPAP use and treatment expectations is essential for effectively guiding patients. Providing anticipatory guidance to address common challenges, such as mask discomfort or nasal irritation, prepares patients for potential obstacles. Facilitating initial assisted exposure to CPAP, typically guided by a trained professional, helps patients become comfortable with the device. Engaging support persons, such as spouses or bed partners, during early education and exposure, fosters a supportive environment. Opportunities for interaction with other CPAP users can offer valuable peer insights and encouragement. Implementing frequent follow-ups in the initial weeks of treatment, coupled with accessible resources for problem-solving, ensures ongoing support. Regular clinical follow-up with a sleep team further reinforces adherence and promotes optimal therapeutic outcomes [26]. The high adherence rate (80.9%) in our study may be attributed to the use of auto-titrating CPAP devices and weekly telemedicine monitoring, which promptly addressed issues such as mask leakage and residual apneas.

The negative predictors of GERD improvement, such as smoking and specific sleep positions, highlight the multifactorial nature of GERD in patients with OSA. Smoking likely exacerbates GERD by increasing acid production and impairing esophageal motility [27], whereas the right-side and supine positions facilitate reflux owing to gravitational effects [24]. The paradoxical association between a higher ODI and worsening GERD symptoms may reflect a subset of patients with more severe nocturnal hypoxia, which could exacerbate esophageal inflammation, as noted in prior studies linking hypoxia to systemic inflammation [28].

Clinical implications

Our findings have several clinical implications. First, adequate CPAP adherence should be emphasized in patients with coexisting OSA and GERD, as it significantly improves both the conditions. Clinicians should prioritize patient education and telemedicine support to enhance adherence, particularly in the first month of therapy, when habit formation is critical. Second, addressing modifiable risk factors, such as smoking cessation and advising against right-side or supine sleep, could enhance GERD outcomes. Third, the direct effect of CPAP on GERD symptoms suggests that CPAP should be considered as a primary therapy in patients with both conditions, even if OSA severity does not fully mediate GERD improvement. Finally, the use of validated tools such as the GERD-Q and ESS in clinical practice can provide objective measures of treatment response, facilitating personalized management.

Limitations

This study has several limitations. The non-randomized observational design limits causal inference, as the lack of a control group (such as sham-CPAP) precludes definitive attribution of outcomes to CPAP therapy. Ethical constraints prevented randomization; however, future studies could explore alternative designs, such as crossover trials. The one-month follow-up period may not capture long-term effects, as GERD and OSA outcomes may continue to evolve beyond this timeframe. The single-center setting limits generalizability as patient demographics and clinical practices may differ across settings. The exclusion of patients using PPIs may have introduced a selection bias, as these medications are commonly used in GERD management. Finally, self-reported adherence to lifestyle modifications (such as diet and smoking cessation) may be subject to recall bias despite efforts to monitor compliance.

## Conclusions

In conclusion, this prospective cohort study demonstrated that adequate CPAP adherence significantly reduced GERD symptom severity and improved OSA severity in patients with coexisting moderate-to-severe OSA and GERD over a one-month period. The direct effect of CPAP on GERD symptoms, independent of changes in OSA severity or daytime sleepiness, underscores its therapeutic value in the management of both conditions. Notably, no adverse effects related to CPAP use such as nasal irritation or mask discomfort were reported. However, smoking and right-sided or supine sleep positions were identified as negative predictors of GERD improvement, highlighting the need for targeted lifestyle interventions. These findings advocate the integration of comprehensive patient education, telemedicine follow-up, and early support strategies to optimize CPAP adherence and achieve favorable outcomes in patients with OSA and GERD.
